# Ethical leadership and job crafting: the role of intrinsic motivation and job feedback

**DOI:** 10.3389/fpsyg.2026.1817186

**Published:** 2026-10-16

**Authors:** Pei Wang, Yu Zhang, Kun Yu

**Affiliations:** 1 School of Labor and Human Resources, Renmin University of China, Beijing, China; 2 China Institute for Human Capital Audit, Renmin University of China, Beijing, China

**Keywords:** cognitive evaluation theory, ethical leadership, intrinsic motivation, job crafting, job feedback, self-determination theory

## Abstract

**Introduction:**

Drawing on Self-Determination Theory (SDT) and its sub-theory Cognitive Evaluation Theory (CET), the present study examines how ethical leadership relates to employees’ job crafting through intrinsic motivation, and how this indirect relationship is contingent on job feedback. We proposed that ethical leadership is positively related to job crafting via intrinsic motivation, and that job feedback moderates this indirect effect by providing competence-relevant informational cues.

**Methods:**

We tested our hypotheses using two-wave data collected from 102 employees in the customer service department of a gaming company in northern China.

**Results:**

The results provided preliminary evidence of a moderated mediation pattern, suggesting that the indirect association between ethical leadership and job crafting through intrinsic motivation varied as a function of job feedback.

**Discussion:**

These findings extend research on ethical leadership and job crafting by clarifying intrinsic motivation as the motivational mechanism and job feedback as a key boundary condition. The theoretical and practical implications are discussed.

## 1 Introduction

As workforces grow more heterogeneous and work itself becomes less predictable, designing a single job that fits every employee is increasingly difficult. Organizations therefore need flexible roles that allow employees to proactively shape their own tasks and responsibilities (Zhang and Parker, 2019). Job crafting refers to these self-initiated actions taken by employees to modify their roles, aligning them with personal needs while simultaneously advancing organizational objectives (Tims et al., 2012; Wrzesniewski and Dutton, 2001). This concept has garnered increasing attention from management scholars due to its proven positive impact on employee outcomes, such as enhanced job satisfaction, well-being, and work performance, as well as organizational benefits, including improved team and organizational performance (Wang et al., 2020). Even in teleworking, job crafting has been shown to enhance remote job performance and act as a buffer against burnout and the negative effects of work overload during the pandemic (Liu et al., 2021), and in remote settings intrinsic motivation has been identified as the mechanism through which job crafting sustains work engagement (Higuchi, 2025).

Previous research on job crafting has predominantly focused on three factors: individual differences (e.g., personality, self-efficacy), job characteristics (e.g., job autonomy, job demands), and demographics (e.g., age, tenure, hours worked) (Lee and Lee, 2018), with meta-analytic evidence corroborating the dispositional and job-characteristic correlates of job crafting while leaving its leadership-related antecedents comparatively underexamined (Rudolph et al., 2017). Recent studies have increasingly shifted attention toward situational antecedents, particularly the influence of leadership on job crafting (Wang et al., 2020). For instance, authentic leadership, empowering leadership (Tang et al., 2020), servant leadership, and transformational leadership have all been shown to positively influence employees’ engagement in job crafting (Wang et al., 2020); these and related leadership forms have further been linked to workplace ethical cultures and employee well-being (Zhu, 2025a, 2025b). Recent studies have begun to examine ethical leadership’s link with employee proactive behaviors (Asif et al., 2023; Abuzaid et al., 2024; Oprea et al., 2025); however, the existing literature lacks systematic elaboration on how ethical leadership shapes job crafting through intrinsic-motivational mechanisms.

This study makes three theoretical and one practical contribution. First, we identify job feedback as a theoretically grounded boundary condition that moderates the indirect effect of ethical leadership on job crafting via intrinsic motivation. Drawing on the Cognitive Evaluation Theory (CET) distinction between feedback’s informational and controlling functions, we propose that informational job feedback may strengthen the autonomy-supportive and competence-affirming signals conveyed by ethical leadership and amplify its motivational impact. This boundary-condition perspective extends prior research that has primarily examined main effects of ethical leadership on proactive behaviors (Asif et al., 2023; Abuzaid et al., 2024; Oprea et al., 2025) and clarifies when ethical leadership most effectively translates into employee-driven role redesign. Second, by integrating Self-Determination Theory (SDT) and its sub-theory CET, we offer a coherent theoretical framework that captures both why (motivational mechanism) and when (boundary condition) ethical leadership shapes job crafting and extends the application of CET to organizational contexts. Third, we provide additional empirical evidence on the mediating role of intrinsic motivation in linking ethical leadership to job crafting, consistent with and extending prior findings on motivational mechanisms (Tu and Lu, 2013; Shareef and Atan, 2019).

Beyond these theoretical advances, our findings offer practical implications for organizations seeking to promote job crafting. The combined effects of ethical leadership and job feedback suggest that human resource management interventions should target both leadership development and feedback systems concurrently rather than in isolation. Organizations can encourage job crafting by building ethical leadership through targeted leadership development programs, improving performance feedback systems to deliver timely competence-relevant information, and aligning these efforts to activate employees’ intrinsic motivation. Such intrinsically motivated job crafting represents a more sustainable form of proactive behavior (Parker et al., 2010) because it is driven by inherent satisfaction and enjoyment rather than by external rewards.

## 2 Theory and hypotheses

### 2.1 Ethical leadership and intrinsic motivation

Ethical leadership is defined as “the demonstration of normative appropriate conduct through personal actions and interpersonal relationships, and the promotion of such conduct to followers through two-way communication, reinforcement, and decision-making” (Brown et al., 2005). Ethical leadership comprises two core dimensions: the ethical person component, wherein ethical leaders possess personal traits such as integrity, honesty, and trustworthiness; and the ethical manager component, in which ethical leaders proactively influence followers’ ethical conduct by encouraging normative behavior and discouraging unethical behavior. Several empirical studies have examined ethical leadership’s positive effects on employee and organizational outcomes, including proactive helpful behavior (Brown et al., 2005), innovative work behaviors (Tu and Lu, 2013), and work engagement (Yang et al., 2020).

Intrinsic motivation refers to engaging in an activity because it is inherently interesting or enjoyable (Ryan and Deci, 2000). Drawing upon Self-Determination Theory (SDT; Deci and Ryan, 1985, 2000; Ryan and Deci, 2000), we propose that ethical leadership enhances employees’ intrinsic motivation by satisfying three basic psychological needs: competence, autonomy, and relatedness. First, ethical leaders care for subordinates’ developmental needs and provide growth-supportive guidance (Brown et al., 2005), which enhances followers’ perceived competence and self-efficacy in pursuing job goals (Walumbwa et al., 2011). Second, ethical leaders give employees voice in decision-making and listen to their opinions with respect (Brown and Treviño, 2006), satisfying followers’ autonomy needs and supporting self-determination (Shareef and Atan, 2019). Third, ethical leaders’ integrity, honesty, and altruistic conduct build relational trust (Mayer et al., 2009), satisfying followers’ relatedness needs and providing the psychological safety required to pursue intrinsically meaningful activities (Walumbwa and Schaubroeck, 2009). The satisfaction of these three needs constitutes the theoretical mechanism specified by SDT through which ethical leadership shapes intrinsic motivation (Tu and Lu, 2016). This needs-satisfaction process was not assessed as a measured variable in the present study but provides the conceptual basis for our predictions within SDT. Hence, we hypothesize:

*Hypothesis 1*: Ethical leadership is positively related to intrinsic motivation.

### 2.2 Intrinsic motivation and job crafting

Job crafting is a self-initiated form of work behavior. Based on the job demands-resources model (JD-R), researchers believed that in job crafting, employees mobilize efforts and competencies to enhance structural and social job resources, increase challenging job demands, as well as decrease hindering job demands (Tims et al., 2013). Enhancing structural resources refers to proactively mobilizing job resources such as opportunities for development, autonomy, or skill variety while enhancing social resources refers to seeking social support, supervisory coaching, or performance feedback (Tuan, 2018). Challenge job demands are viewed as demands that have the potential to promote mastery and future gains, while hindrance job demands are perceived as constraints that impede progress (Tuan, 2018).

Intrinsic motivation, defined as engaging in activities for their inherent satisfaction and enjoyment (Ryan and Deci, 2000), is the motivational energy that drives employees to go beyond formal role requirements. When intrinsically motivated, employees experience a deep interest in their work and invest discretionary effort to proactively reshape job characteristics. This motivational state is particularly conducive to job crafting because it directs employees’ attention toward how tasks, relationships, and cognitions could be reconfigured to better align with personal interests and competencies (Wrzesniewski and Dutton, 2001; Tims et al., 2012). Empirical evidence consistently supports the link that intrinsic motivation has been positively associated with proactive behaviors including job crafting, innovation, and engagement (Niessen et al., 2016; Bindl et al., 2019), and studies focusing specifically on motivated role redesign show that intrinsically motivated employees are more likely to engage in job crafting (Lee and Song, 2019) and, in particular, in cognitive crafting that reframes the meaning of their work (Kilic and Kitapci, 2023). Within SDT, intrinsic motivation arises from the satisfaction of competence, autonomy, and relatedness needs; we treat this needs-satisfaction process as the conceptual underpinning of intrinsic motivation rather than as a separately measured construct in our model. Therefore, we hypothesize:

Hypothesis 2: Intrinsic motivation is positively related to job crafting.

Beyond these separate associations, we expect intrinsic motivation to be the motivational mechanism that links ethical leadership to job crafting. By treating employees fairly, modeling ethical conduct, and offering principled guidance, ethical leaders create an autonomy-supportive context that satisfies employees’ needs for competence, autonomy, and relatedness and, in turn, strengthen their intrinsic motivation (Hypothesis 1); intrinsically motivated employees then proactively reshape their tasks, relationships, and cognitions (Hypothesis 2). Although ethical leadership has been associated with a range of employee proactive behaviors (Asif et al., 2023; Abuzaid et al., 2024), the motivational pathway through which it shapes job crafting has received limited empirical attention. Integrating these two links, we propose that ethical leadership relates to job crafting primarily through the intervening motivational state of intrinsic motivation rather than through a direct path. Therefore, we hypothesize:

Hypothesis 3: Intrinsic motivation mediates the relationship between ethical leadership and job crafting.

### 2.3 The moderating role of job feedback

Cognitive Evaluation Theory (CET; Deci et al., 1975), as a sub-theory of SDT, distinguishes two functional aspects of feedback. Feedback can influence intrinsic motivation through informational and controlling functions: when perceived as providing competence information, it satisfies the need for competence and enhances intrinsic motivation; when perceived as controlling behavioral outcomes, it may undermine autonomy and intrinsic motivation (Cusella, 1984; Jussim et al., 1992; Kluger and DeNisi, 1996).

Although feedback can serve either function, scholars have argued that task-performance feedback delivered as descriptive information about how well employees are doing, rather than as a contingent reward or sanction, is generally informational in nature (Kluger and DeNisi, 1996). When such informational feedback is present, it complements ethical leadership in a theoretically meaningful way. Ethical leaders convey autonomy-supportive signals through fair treatment, role modeling, and ethical guidance (Brown et al., 2005), but these signals require corroborating competence information to be fully translated into sustained intrinsic motivation. When job feedback is high, employees obtain frequent information about their competence and behavioral effectiveness, which validates and amplifies the autonomy support and growth opportunity signals conveyed by ethical leaders. Job feedback and ethical leadership thus form a complementary fit, and together they help satisfy the three basic psychological needs (competence, autonomy, and relatedness) that generate intrinsic motivation (Deci and Ryan, 2000; Gagné and Deci, 2005). When job feedback is low, ethical leaders’ positive signals lack confirmatory information, weakening their motivational impact.

Hypothesis 4: Job feedback moderates the relationship between ethical leadership and intrinsic motivation, such that the relationship is stronger when job feedback is higher.

Integrating the mediation pathway proposed in Hypothesis 3 with the moderation logic of Hypothesis 4, we expect job feedback to also moderate the indirect effect of ethical leadership on job crafting through intrinsic motivation. When job feedback is high, ethical leadership more strongly activates intrinsic motivation, which in turn promotes job crafting; when job feedback is low, the indirect effect weakens because the motivational mechanism is less fully engaged. This moderated mediation pattern reflects the integrated SDT-CET framework, in which informational feedback functions as a boundary condition for the motivational mechanism through which ethical leadership shapes proactive role behavior.

Hypothesis 5: Job feedback moderates the indirect effect of ethical leadership on job crafting via intrinsic motivation, such that the indirect effect is stronger when job feedback is higher.

We summarize the proposed model in Figure 1.

**Figure 1:**
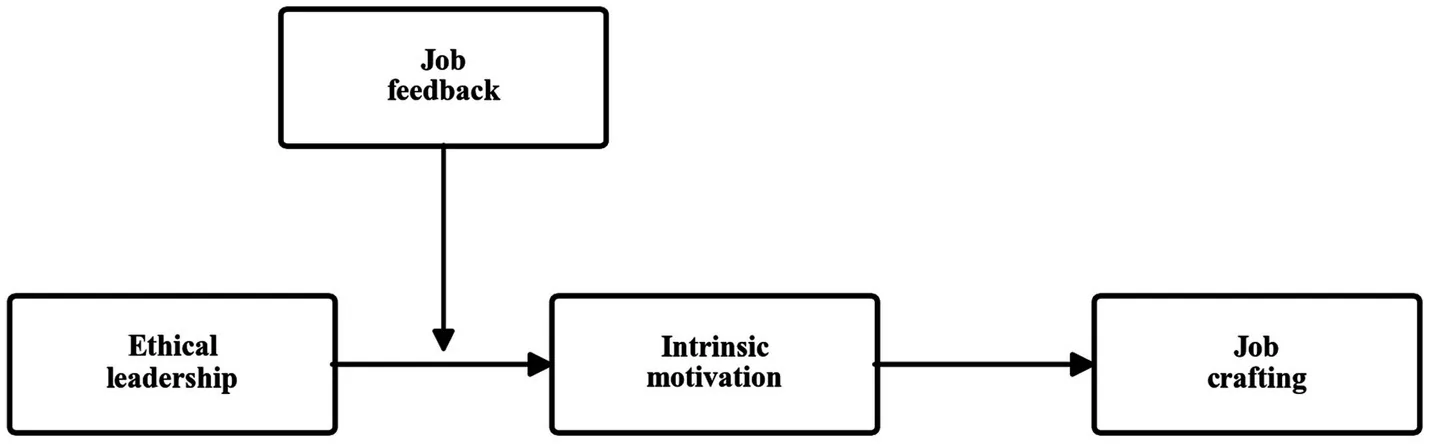
Hypothesized research model.

## 3 Method

### 3.1 Participants and procedures

Data for this study were collected from the customer service department of a gaming company located in a city in northern China during 2021. At Time 1, a total of 176 employees agreed to participate. At Time 1, participants received a survey package containing a letter outlining the study’s purpose and instructions, along with a questionnaire assessing ethical leadership, baseline job crafting, and demographic information. At Time 2, 1 month later, participants received another questionnaire evaluating job feedback, intrinsic motivation, and job crafting.

Of the 176 employees, 102 completed both waves of the survey, resulting in a response rate of 58%. Multiple logistic regression analysis showed that none of the demographic variables significantly predicted survey completion (*p* > 0.10 for all). Among the 102 participants, 55 (53.9%) were male. At Time 1, the average age of participants was 25.71 years (*SD* = 5.54), and their average organizational tenure was 36.20 months (*SD* = 34.45). Half of the participants held a bachelor’s degree or higher (50%).

To reduce the risk of common method variance, we adopted several procedural remedies in the original data collection: (a) all responses were anonymous and confidential; (b) item order was randomized within each scale to reduce response set effects; (c) predictor and criterion variables were temporally separated by approximately 1 month (Time 1 vs. Time 2 measurement); and (d) we explicitly emphasized to respondents that there were no right or wrong answers, encouraging candid reporting (Podsakoff et al., 2012).

All measures were translated from English to Chinese using Brislin’s (1980) translation–back-translation procedure. Two bilingual researchers independently translated the original items into Chinese, and a third bilingual researcher back-translated the Chinese version into English. Discrepancies between the original and back-translated versions were resolved through discussion among the translators to ensure conceptual equivalence.

### 3.2 Measures

Unless otherwise noted, participants were asked to indicate the extent to which they agreed with each item based on their experiences at work on a 7-point Likert scale (from 1 = “Strongly disagree” to 7 = “Strongly agree”).

Ethical leadership was measured by the 10-item ethical leadership scale (Brown et al., 2005). Respondents rated the items on a 7-point scale (1 = highly unlikely, 7 = highly likely). A sample item is ‘My supervisor makes fair and balanced decisions’. Higher scores represent higher levels of ethical leadership. Cronbach’s *α* for ethical leadership in this study was 0.95.

Job feedback was measured by the 3-item job feedback scale adapted from Gonzalez-Mulé et al. (2016). Respondents rated the items on a 7-point scale (1 = strongly disagree, 7 = strongly agree). A sample item is ‘Leaders or colleagues usually give feedback on the quality of work I have done’. Higher scores represent higher levels of job feedback. Cronbach’s α for job feedback in this study was 0.90.

Intrinsic motivation was measured by the 3-item intrinsic motivation dimension of the work motivation scale (Gagné et al., 2010). Respondents rated the items on a 7-point scale (1 = not at all, 7 = exactly). A sample item is ‘Because I enjoy this work very much’. Higher scores represent higher levels of intrinsic motivation. Cronbach’s α for intrinsic motivation in this study was 0.94.

Job crafting was measured by a 13-item job crafting scale adapted from Petrou et al. (2012). Respondents rated the items on a 7-point scale (1 = strongly disagree, 7 = strongly agree). A sample item is “I have asked for more tasks if I finish my work.” Higher scores represent higher levels of job crafting. Cronbach’s αs for job crafting were 0.84 at Time 1 (baseline) and 0.92 at Time 2.

*Control* var*iables*. Employees’ gender (1 = male, 2 = female), age (in years), educational level (1 = junior college or below, 2 = bachelor’s degree, 3 = master’s degree or above), organizational tenure (in months) were controlled in the current study.

Given the complexity of the moderated mediation model and the modest sample size, we adopted item parceling for the job crafting scale following recommendations by Little et al. (2002). Because job crafting was modeled as a single latent construct in the present analysis, we formed three parcels by assigning the 13 items into three groups (items 1–4, 5–8, and 9–13), each serving as a balanced indicator of the overall job crafting factor. This procedure reduces the number of estimated parameters and improves the indicator-to-sample-size ratio, yielding more stable estimates in moderate samples; it is an estimation choice and does not represent the substantive subdimensions of job crafting. We acknowledge that parceling can mask within-construct multidimensionality (Bandalos and Finney, 2001) and therefore interpret the measurement model with appropriate caution. Other constructs (ethical leadership with 10 items, job feedback with 3 items, intrinsic motivation with 3 items) used individual items as indicators without parceling.

### 3.3 Analytical strategy

We tested the hypothesized moderated mediation model using Hayes (2018) PROCESS Model 7, in which job feedback (W) moderates the X → M path from ethical leadership to intrinsic motivation, with intrinsic motivation as the mediator linking ethical leadership to job crafting. All continuous predictors (ethical leadership, job feedback, and baseline job crafting) were mean-centered prior to forming the interaction term to facilitate interpretation of main effects (Aiken and West, 1991). Models were estimated by ordinary least squares regression using Python (statsmodels 0.14), with 95% bootstrap confidence intervals based on 5,000 resamples for conditional indirect effects.

## 4 Results

The means, standard deviations, reliabilities, and zero-order correlations among the focal variables are presented in Table 1.

**Table 1:** Means, standard deviations, reliabilities, and correlations.

Variable	*M*	*SD*	1	2	3	4	5	6	7	8	9
1. Gender	1.46	0.50	—								
2. Age	25.71	5.54	−0.00	—							
3. Education	2.47	0.59	−0.04	0.12	—						
4. Tenure	36.20	34.45	0.02	0.40***	−0.00	—					
5. Baseline JC	5.96	1.02	−0.26**	−0.13	−0.03	−0.15	(0.84)				
6. EL	5.91	1.02	−0.23*	−0.11	−0.05	−0.13	0.93***	(0.95)			
7. JFB	5.23	1.21	−0.04	−0.27**	−0.19	−0.09	0.52***	0.52***	(0.90)		
8. INM	5.24	1.29	−0.12	−0.14	−0.10	−0.05	0.53***	0.49***	0.69***	(0.94)	
9. JC	5.42	0.90	−0.12	−0.28**	−0.11	−0.15	0.59***	0.56***	0.76***	0.73***	(0.92)

*N* = 102. Cronbach’s alpha coefficients are reported in parentheses along the diagonal. 1 = male, 2 = female. T1 = Time 1; T2 = Time 2. *p < 0.05, **p < 0.01, ***p < 0.001.

### 4.1 Tests of the measurement model

We conducted confirmatory factor analysis (CFA) in Python (semopy) to examine whether the constructs in the current research were empirically distinguishable. The measurement model included five latent variables: Time 1 baseline job crafting, Time 1 ethical leadership, Time 2 job feedback, Time 2 intrinsic motivation, and Time 2 job crafting. The five-factor measurement model showed acceptable fit, χ^2^ (199) = 358.21, *p* < 0.001, Comparative Fit Index (CFI) = 0.928, Tucker–Lewis Index (TLI) = 0.916, Root Mean Square Error of Approximation (RMSEA) = 0.089. We note that the RMSEA value of 0.089 slightly exceeds the conventional threshold of 0.080 (Hu and Bentler, 1999), indicating that fit was at the boundary of acceptability rather than uniformly excellent; we have therefore avoided characterizing the fit as “good” and instead interpret the model with appropriate caution. We compared the hypothesized five-factor model against a model in which constructs measured at the same time point were combined as a single factor; the five-factor model provided a better fit, supporting the discriminability of the focal constructs.

To statistically assess the potential influence of common method variance (CMV), we adopted the common method factor (CMF) approach recommended by Williams and Anderson (1994), which has largely supplanted the now-criticized Harman’s single-factor test. Specifically, on top of the five-factor measurement model, we added a latent method factor on which all indicators simultaneously loaded along with their substantive constructs. The inclusion of CMF produced a non-trivial improvement in model fit (ΔCFI = +0.016, exceeding the 0.01 ΔCFI cutoff recommended by Cheung and Rensvold, 2002; ΔRMSEA = −0.006), suggesting that common method variance may have some influence on the parameter estimates. We therefore explicitly identify CMV as the foremost limitation of this study in the Limitations section and call for future research using supervisor–subordinate matched dyads.

### 4.2 Tests of the hypothesized model

We first estimated the moderated mediation model (Hayes, 2018 PROCESS Model 7) by regressing intrinsic motivation on ethical leadership, job feedback, and their interaction, controlling for gender, age, educational level, tenure, and baseline job crafting. The unstandardized estimates predicting intrinsic motivation (Time 2) are presented in Table 2; estimates predicting job crafting (Time 2) are presented in Table 2.

**Table 2:** Regression results.

Variable	Intrinsic motivation	Job crafting
Intercept	5.103** (0.641)	6.139** (0.395)
Gender	−0.110 (0.188)	0.026 (0.118)
Age	0.009 (0.019)	−0.024* (0.011)
Educational level	−0.023 (0.158)	−0.045 (0.098)
Tenure	0.000 (0.003)	−0.001 (0.002)
Baseline job crafting (centered)	0.390 (0.243)	0.193 (0.155)
Ethical leadership (centered)	0.010 (0.249)	0.050 (0.150)
Job feedback (centered)	0.591** (0.094)	—
Ethical leadership × Job feedback	0.155* (0.072)	—
Intrinsic motivation (centered)	—	0.394** (0.052)
R^2^	0.543	0.623
*F*	13.82*** (8, 93)	22.23*** (7, 94)
*N*	102	102

*N* = 102. Entries are unstandardized regression coefficients with standard errors in parentheses. All continuous predictors are mean-centered. The em-dash (—) denotes a variable not included in that regression. *p < 0.05; **p < 0.01; ***p < 0.001 (two-tailed test).

The results from the mediation model showed that ethical leadership (centered) was not associated with intrinsic motivation as an unconditional main effect (b = 0.010, *p* > 0.05), but its effect was qualified by a significant interaction with job feedback (b = 0.155, SE = 0.072, t = 2.16, *p* < 0.05, 95% CI [0.013, 0.298]), which indicates that the motivational benefits of ethical leadership emerge contingent on the presence of informational feedback. Examining the outcome regression (Table 2), intrinsic motivation was positively related to job crafting (b = 0.394, *p* < 0.001), consistent with its role as a proximal motivational mechanism for employees’ role redesign. The direct effect of ethical leadership on job crafting was not significant after accounting for intrinsic motivation (b = 0.050, *p* > 0.05). Because the ethical leadership–intrinsic motivation (a) path was itself conditional on job feedback rather than significant on average, we did not interpret these results as unconditional (full) mediation; in this first-stage moderated mediation model (Hayes, 2018, PROCESS Model 7) the relevant test is the index of moderated mediation rather than a single unconditional indirect effect. Hypothesis 1 posited an unconditional positive ethical leadership–intrinsic motivation relationship; the non-significant main effect (b = 0.010, *p* > 0.05) does not support H1. Although the conditional slope of ethical leadership on intrinsic motivation was positive at high job feedback (+1 SD; simple slope = +0.198) and negative at low job feedback (−1 SD; simple slope = −0.179), neither conditional slope was individually significant (*p* = 0.49 and *p* = 0.46, respectively); support for H4 therefore rests on the significant interaction term (b = 0.155, *p* = 0.033). Hypothesis 2 (the intrinsic motivation–job crafting link) was supported, and Hypothesis 3 (mediation via intrinsic motivation) was supported only conditionally, operating where job feedback is high enough to render the a path positive, rather than as a general unconditional mechanism; the moderated indirect effect is examined below (H5).

To follow up on Hypothesis 4, we probed the EL × JFB interaction at ±1 SD of job feedback, as illustrated in Figure 2. The centered conditional slope of ethical leadership on intrinsic motivation was +0.198 (SE = 0.285, *p* = 0.49) at high feedback (+1 SD) and −0.179 (SE = 0.241, *p* = 0.46) at low feedback (−1 SD). Although neither simple slope was individually significant, their difference, captured by the significant interaction term, indicates that job feedback shifts the ethical leadership–intrinsic motivation association in a positive direction, consistent with the integrated CET–SDT prediction that informational feedback enhances the perception of competence and autonomy that ethical leadership signals. The limited precision of these conditional estimates reflects the modest sample size and is addressed in the Limitations section.

**Figure 2:**
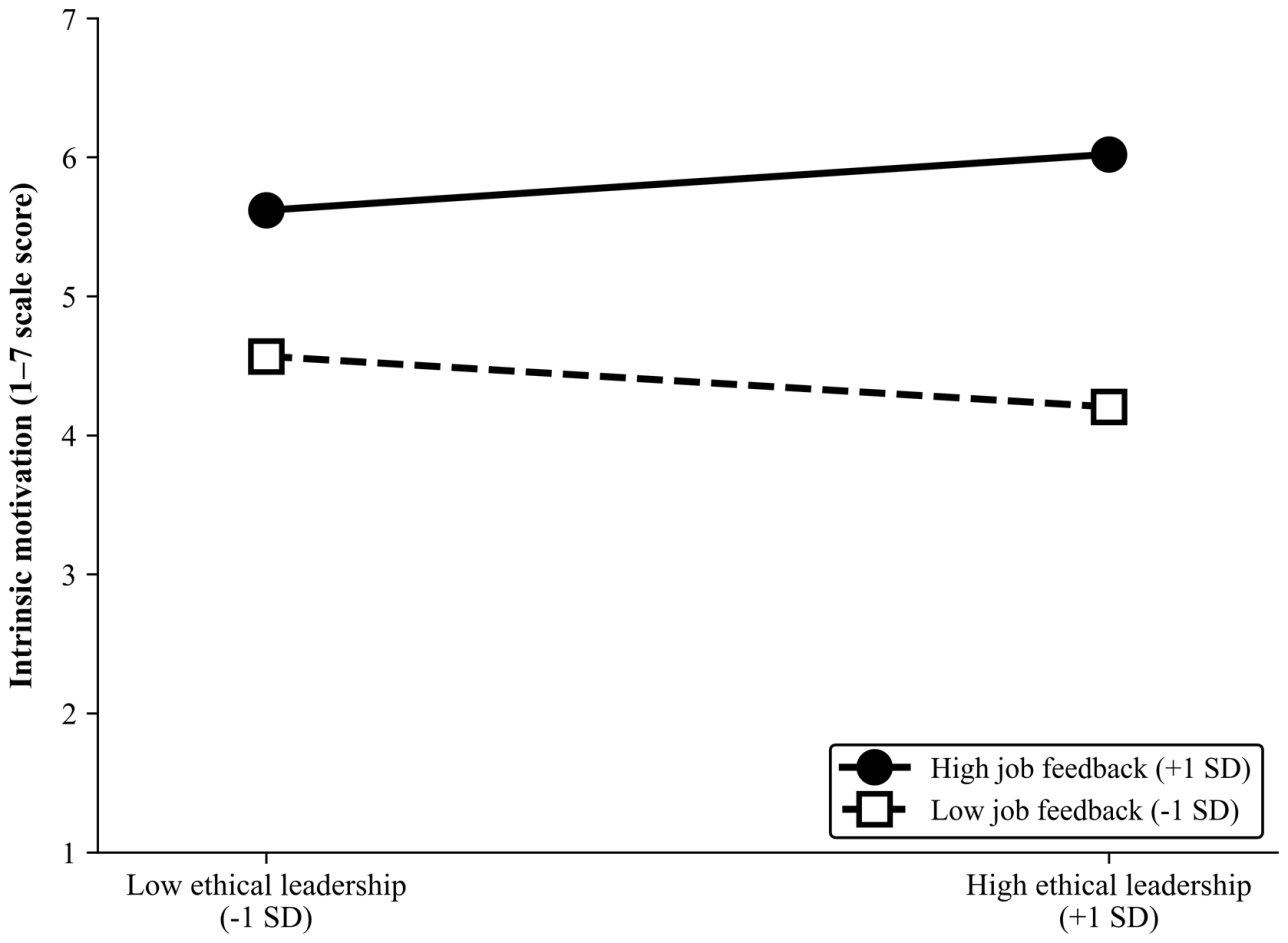
The interaction effect of ethical leadership and job feedback on intrinsic motivation.

To test Hypothesis 5, we estimated conditional indirect effects using 5,000 bias-corrected bootstrap resamples. The Index of Moderated Mediation (Hayes, 2015) was significant (Index = 0.061, 95% bias-corrected bootstrap CI [0.020, 0.143]), indicating that the indirect effect of ethical leadership on job crafting via intrinsic motivation is moderated by job feedback. At high job feedback (+1 SD), the conditional indirect effect was 0.078 (95% CI [−0.092, 0.310]); at low job feedback (−1 SD), the conditional indirect effect was −0.070 (95% CI [−0.247, 0.094]). Although the significant Index confirms that the magnitude of the indirect effect varies systematically across feedback levels, the point-estimate confidence intervals at extreme feedback values (±1 SD) include zero, which reflects limited estimation precision at *N* = 102; this constitutes a power limitation we discuss further in the Limitations section.

## 5 Discussion

Drawing upon Self-Determination Theory (SDT) and its sub-theory Cognitive Evaluation Theory (CET), the present study examines how ethical leadership shapes employees’ job crafting through intrinsic motivation and how this indirect effect is contingent on job feedback. Support for the proposed moderated mediation rested primarily on a significant index of moderated mediation, which indicated that the indirect effect of ethical leadership on job crafting via intrinsic motivation varied systematically with job feedback and became more positive as job feedback increased. The conditional indirect effects estimated at conventional high and low levels of job feedback (±1 SD), however, were imprecisely estimated and their confidence intervals included zero. We therefore interpret these findings as preliminary evidence that informational feedback amplifies the motivational benefits of ethical leadership, rather than as conclusive support for the indirect effect at specific levels of job feedback.

The current study contributes to theory and practice in several ways. First, our results identify informational job feedback as a boundary condition for the motivational pathway from ethical leadership to job crafting. This pattern moves beyond the main-effect focus of earlier work on ethical leadership and proactive behavior (Asif et al., 2023; Abuzaid et al., 2024; Oprea et al., 2025) and points to a situational contingency that ethical leadership translates into employee-driven role redesign most readily when employees also receive competence-relevant feedback.

Second, our framework integrates Self-Determination Theory and its sub-theory CET into a single moderated mediation logic, joining the proximal motivational mechanism (intrinsic motivation) with its contextual moderator (informational feedback). Prior leadership research has often drawn on SDT-based motivational arguments without specifying the feedback conditions under which leadership effects unfold; our account makes that condition explicit. It also shows that CET, originally formulated to explain how extrinsic incentives affect intrinsic motivation in experimental settings, can be extended to organizational leadership once the informational and controlling functions of feedback are taken into account.

Third, we add evidence that intrinsic motivation carries the link between ethical leadership and job crafting (Tu and Lu, 2013; Shareef and Atan, 2019). When employees see their leader as ethical, they experience greater autonomy and competence, identify job-related challenges, and redesign their tasks, relationships, and cognitive framing of work; because the work itself becomes engaging, this crafting is driven by interest rather than by external reward. This account brings together scattered findings on ethical leadership and proactive behavior (Brown et al., 2005; Tu and Lu, 2013; Abuzaid et al., 2024) under a single motivational mechanism and helps address the relative neglect of job crafting’s antecedents (Kim and Beehr, 2022).

Our findings also have practical implications. Because predictable, one-size-fits-all job design fits a heterogeneous workforce poorly (Hackman and Oldham, 1976), organizations increasingly rely on employees to shape their own roles (Wrzesniewski and Dutton, 2001), and the growing use of generative AI at work makes this kind of employee-driven crafting more important. Our results suggest that pairing ethical leadership with effective feedback can activate it. Given the positive relationship between ethical leadership and job crafting, managers should adopt an ethical leadership style that respects employees’ dignity, empowers them, and makes their roles more meaningful; doing so can prompt employees to generate new ideas and put them into action (Tu and Lu, 2013).

Because job feedback strengthens this relationship, organizations should prioritize timely, constructive feedback and use multiple channels, such as regular one-on-one meetings, 360-degree feedback systems, and project debriefs, to give employees continuous, high-quality input. Beyond formal performance reviews, more frequent and informal feedback may especially help employees who are inclined to craft their jobs (Kim and Beehr, 2022). Immediate, specific feedback supports continuous improvement. In practical terms, combining ethical leadership development with stronger feedback systems can jointly activate employees’ intrinsic motivation.

## 6 Limitations and future directions

This study has several limitations that should be acknowledged and that warrant further examination in future research.

First, several limitations stem from our research design. Because all variables were self-reported by the same source, common method variance is a concern; although we adopted procedural remedies (anonymity, item randomization, and temporal separation between Time 1 and Time 2) and a statistical common method factor test (ΔCFI = +0.016, exceeding the 0.01 ΔCFI cutoff; Cheung and Rensvold, 2002), we cannot fully rule out its influence, and the unusually high Time 1 correlation between ethical leadership and baseline job crafting (*r* = 0.93) suggests potential same-source method effects (Podsakoff et al., 2003) and should be interpreted with caution. Relatedly, although the two-wave design separated Time 1 baseline measures from Time 2 outcomes, the mediator (intrinsic motivation), moderator (job feedback), and outcome (job crafting) were measured concurrently at Time 2, which precludes strict causal inference and leaves alternative interpretations open. Future research should employ multi-source or multi-wave designs with explicit temporal precedence to disentangle substantive relationships from method-based covariation and to strengthen causal inference.

Second, the modest sample size (*N* = 102) limits statistical power and the precision with which interaction and moderated mediation effects can be estimated; the 95% confidence intervals for the focal effects, though informative, are relatively wide. Rather than relying on *post-hoc* power calculations, which are uninformative for data already collected (Hoenig and Heisey, 2001), we interpret the results with reference to confidence-interval width. In addition, of the 176 participants recruited at Time 1, 74 attrited at Time 2; demographic variables did not significantly predict attrition (all *p* > 0.10), but Time 1 substantive-variable data for the attrited participants were not retained, precluding a full attrition test on the focal variables. Future research should replicate the model with larger samples and retain complete Time 1 data to support attrition analyses.

Third, several measurement issues warrant attention. For the confirmatory factor analysis, job crafting was represented by three sequential item parcels (items 1–4, 5–8, and 9–13) rather than by its individual items; although this reduced the parameter-to-sample-size ratio, parceling can mask within-construct multidimensionality (Bandalos and Finney, 2001), and because we modeled job crafting as a single latent construct we did not separately estimate an item-level multidimensional solution. The unusually high reliabilities for ethical leadership (0.95) and intrinsic motivation (0.94) further suggest possible item redundancy. Moreover, job feedback was assessed with a three-item scale (Gonzalez-Mulé et al., 2016) that captures whether employees receive feedback but does not distinguish whether it is experienced as informational or controlling, a meaningful gap, given that our account rests on the premise that feedback strengthens intrinsic motivation only insofar as it conveys competence-relevant information rather than external control (Ryan, 1982; Cusella, 1984; Kluger and DeNisi, 1996). Future research should adopt shorter, more differentiated scales and measures that separately assess the informational and controlling functions of feedback.

Fourth, the generalizability and theoretical scope of our findings are bounded. Data were collected from a single customer service department within one gaming company in northern China, so the findings may not generalize to other industries, occupations, or cultural contexts. Furthermore, we modeled intrinsic motivation as the single mechanism linking ethical leadership to job crafting, yet other routes, such as self-efficacy and psychological empowerment (Walumbwa et al., 2011; Abuzaid et al., 2024), may operate alongside or in place of it, and ethical leadership is only one of several autonomy-supportive styles (e.g., empowering and servant leadership; Tang et al., 2020) that promote job crafting. Because we measured neither the alternative mediators nor the alternative leadership styles, future research should estimate these pathways jointly and assess the incremental contribution of ethical leadership and intrinsic motivation over and above these adjacent mechanisms and styles.

Building on these limitations, future research should adopt three-wave designs with explicit temporal precedence to strengthen causal inferences, use supervisor-subordinate matched data to reduce common method variance, expand samples beyond a single industry and culture to enhance external validity, and distinguish between informational and controlling forms of feedback in line with cognitive evaluation theory.

## 7 Conclusion

Job crafting contributes to organizational success (Tims and Bakker, 2010). Through the activation of subordinates’ intrinsic motivation, ethical leaders could encourage bottom-up job-crafting behaviors, a process that is strengthened when employees receive timely and meaningful job feedback. Collectively, these findings provide preliminary evidence on how ethical leadership relates to job crafting and offer initial practical guidance for promoting job crafting in the workplace, pending replication with larger samples and stronger causal designs.

## Data Availability

The raw data supporting the conclusions of this article will be made available by the authors, without undue reservation.
